# Predictors of enrollment in a virtual diabetes prevention program among women veterans: a retrospective analysis

**DOI:** 10.1186/s12905-024-03314-6

**Published:** 2024-08-24

**Authors:** Allyson Malone, Kimberly Clair, Catherine Chanfreau, Bevanne Bean-Mayberry, Rebecca Oberman, Rachel Lesser, Cody Knight, Erin Finley, Alison Hamilton, Melissa M. Farmer, Tannaz Moin

**Affiliations:** 1 David Geffen School of Medicine at UCLA, Los Angeles, CA USA; 2 Center for the Study of Healthcare Innovation, Implementation & Policy (CSHIIP), Greater Los Angeles Healthcare System, Los Angeles, VA, CA USA; 3 VA Informatics and Computing Infrastructure (VINCI), VA Salt Lake City Healthcare System, Salt Lake City, UT USA; 4 University of Texas Health Science Center, San Antonio, TX USA

**Keywords:** Diabetes prevention, Women’s health, Implementation science, Remote healthcare delivery, Veterans

## Abstract

**Background:**

The Diabetes Prevention Program (DPP) is a nationally disseminated lifestyle intervention shown to prevent type 2 diabetes (diabetes). However, enrollment in the program remains variable. We sought to identify patient characteristics associated with enrollment in a virtual DPP program among women Veterans to inform ongoing diabetes prevention efforts.

**Methods:**

We conducted a retrospective analysis of 2021–2024 Department of Veterans Affairs (VA) data collected through the VA Enhancing Mental and Physical Health of Women through Engagement and Retention (EMPOWER) 2.0 Program, an effectiveness-implementation trial to expand access to preventative health services for women Veterans. We included women meeting DPP eligibility criteria (BMI ≥ 25 kg/m^2^ [or ≥ 23 if Asian] with *≥* 1 risk factor for diabetes [e.g., prediabetes]) who received care at six VA sites implementing virtual DPP. We used logistic regression to examine the association between DPP enrollment and prior use of VA preventive services for weight management or diabetes prevention including the VA MOVE! clinic, Whole Health visits, nutrition visits, weight loss medications, and/or metformin. We adjusted for sociodemographic factors, comorbidities, number of DPP recruitment contacts, and site.

**Results:**

A total of 1473 women Veterans received DPP outreach. On average, their age was 53 years (range 20–96), BMI 34 kg/m^2^, HbA1c 5.9%, 0.7% were Asian, 44% Black, 2% Hispanic, and 44% White. In our adjusted models, prior use of VA preventative services was not significantly associated with DPP enrollment. Younger women (OR:0.97, *p* = 0.002) and those who received more recruitment contacts (OR:2.63, *p* < 0.001), were significantly more likely to enroll in DPP. Women with housing instability were significantly less likely to enroll (OR:0.44, *p* = 0.029).

**Conclusions:**

We found no difference in women Veterans’ enrollment in DPP based on prior use of VA weight management and prevention services. Frequency of outreach by VA sites may increase engagement in lifestyle interventions. Virtual DPP may support engagement in preventive lifestyle interventions for diverse groups of women Veterans, as a first program or as a complement to other VA services.

**Trial registration:**

ClinicalTrials.gov, NCT05050266. Registered on 20 September 2021.

**Supplementary Information:**

The online version contains supplementary material available at 10.1186/s12905-024-03314-6.

## Introduction

Prediabetes affects approximately 98 million adults in the United States and increases risk of incident type 2 diabetes (T2D) and associated co-morbidities including chronic kidney disease and neuropathy [1, 2]. The National Diabetes Prevention Program (DPP) is a Centers for Disease Control and Prevention (CDC)-led initiative to increase access to an evidence-based intensive lifestyle intervention shown to reduce incident T2D risk among adults with prediabetes and overweight/obesity. Studies have shown that the DPP is effective for weight loss and lowering incident T2D risk even after 15 years follow-up [3, 4].

The National DPP was originally delivered as a one-year, in-person program. The original DPP intervention included a 16-lesson curriculum delivered in-person on a one-to-one basis by trained staff over 24 weeks [5]. The DPP was later adapted for group-based delivery but in-person delivery posed barriers for some participants. Specifically, travel to in-person sessions created difficulties completing the program [6]. Development of online or virtual delivery of DPP helped mitigate some of the barriers related to in-person attendance and was also found to be effective for weight loss. For example, a randomized control trial with 599 online DPP participants showed a significant reduction in hemoglobin A1c (HbA1c) and weight loss among online DPP participants versus enhanced standard of care after one year, though enhanced standard of care in this study was defined as a single small-group diabetes prevention class [7]. A comparative effectiveness study across multiple VA sites also showed higher participation rates with virtual as compared to in-person DPP but similar weight loss at 12-months follow-up [8].

While both virtual and in-person DPP have been shown to be effective, recruitment and enrollment of participants remain a challenge. One study found that out of 2341 patients with prediabetes, only 4.2% had ever been referred to DPP and only 2.4% had participated [9]. Women face additional barriers to lifestyle intervention program enrollment when compared to men, such as competing demands for time and lack of prediabetes awareness [10, 11]. Additionally, though incidence of T2D is declining in the general population, prediabetes and T2D prevalence is increasing in the premenopausal female population [11]. Women of child-bearing age have also been less likely to engage in an in-person DPP than older women [12]. These findings suggest an opportunity to increase education and awareness of lifestyle interventions for T2D prevention, especially among women. This is particularly important for women Veterans who receive care in the Veterans Health Administration (VA) who often have higher rates of T2D and other chronic conditions than the general female population [13].

Women Veterans may also face additional barriers to receiving in-person care, including perceived safety in male-dominated VA settings and variable degrees of gender sensitivity among VA staff and providers [14]. In prior VA translational DPP studies, women Veterans perceived virtual DPP as convenient and flexible [8, 15], suggesting that virtual DPP is a promising modality to address T2D risk in this population. Studies outside the VA have shown that DPP enrollment may be higher in women as compared to men, and higher in older participants as compared to younger participants [16, 17]. However, to our knowledge no study has examined factors that may be associated with virtual DPP enrollment among women Veterans. Thus, our objective was to identify patient-level factors associated with virtual DPP enrollment among women Veterans from multiple VA sites implementing virtual DPP between 2022 and 2024. We hypothesized that women who had previously used VA preventative lifestyle services would be more likely to enroll in DPP than those who had not used the services.

## Methods

We conducted a retrospective analysis of 2021–2024 VA electronic health record (EHR) and administrative data collected as part of the VA Enhancing Mental and Physical Health of Women through Engagement and Retention (EMPOWER) 2.0 Quality Enhancement and Research Initiative (QUERI) program. EMPOWER 2.0 is an ongoing cluster-randomized hybrid type 3 effectiveness-implementation trial to expand access to preventative health services for women Veterans, including a virtual DPP. The details of EMPOWER 2.0 have been described elsewhere [18]. A gender tailored virtual DPP was implemented as part of a quality improvement initiative across multiple VA sites between 2022 and 2024 (EMPOWER 2.0 trial was deemed to be non-research). For this analysis, we included data from the six VA sites that completed virtual DPP implementation between April 2022 and March 2024.

### Study design and populations

EMPOWER 2.0 DPP eligibility criteria included women with body mass index (BMI) ≥ 25 kg/m^2^ (or ≥ 23 if self-reporting Asian race) and at least one additional risk factor for T2D. Additional risk factors included prediabetes (HbA1c 5.7–6.5% or fasting plasma glucose 100–125 mg/dL), history of gestational diabetes (GDM) or high risk score based on the CDC prediabetes risk test [19]. Women with a history of T2D (based on labs, diagnostic codes or use of any antiglycemic medications with the exception of monotherapy with metformin or GLP-1 agonists) and those currently pregnant were excluded. We did not exclude women who used GLP-1 agonists as they are often prescribed in conjunction with lifestyle intervention at the VA and only 2.2% (*N* = 33) women in our cohort had received a GLP-1a prescription for weight management. We restricted the analytic cohort to women who were contacted at least once by their respective site for recruitment into DPP.

We used electronic health record and administrative data from the VA Corporate Data Warehouse (CDW) to identify women who met DPP eligibility criteria at each site. The list of eligible women Veterans from each site was included in a DPP recruitment list that was updated on a monthly basis and available to the local DPP implementation team through a secure VA SharePoint. The DPP recruitment list included the women Veterans’ medical record identification, age, BMI, HbA1c, and urban/rural residence, which sites could use to guide their local recruitment and outreach. Sites were also provided recruitment material templates (e.g., flyer and brochure templates). Each VA site determined how they would conduct outreach (e.g., invitation letters, phone calls, point-of-care referrals, etc.) and subsequently documented each contact attempt in the DPP recruitment SharePoint. Once a woman Veteran opted-in for DPP enrollment, they were provided a unique voucher code that they could use to register and enroll with the virtual DPP vendor which was delivered by a CDC-recognized, third-party DPP organization. The virtual DPP vendor tracked participation and weight change data which was provided back to local VA sites to track participant progress.

### Measures

Our main outcome of interest was enrollment in DPP, defined as redeeming the unique voucher and completing enrollment in the virtual DPP platform. Our regressor of interest was a dichotomous indicator of prior use of VA preventative services for weight management or T2D prevention (1 = yes for any versus 0 = none). Use of any of the following within the last three years was included in our composite outcome of interest: (1) MOVE! Program participation, (2) Whole Health clinic visits, (3) nutrition or dietetics visits, (4) use of weight loss medications, and/or (5) use of metformin. Use of these services and medications was defined based on CDW stop codes (i.e., VA administrative and billing codes) for outpatient visits and nutrition/dietetics visits, and prescription medication orders filled. Number of DPP recruitment contacts was obtained from the DPP recruitment SharePoint (where it was documented by site staff in real-time).

We collected baseline patient sociodemographic and clinical characteristics including age, race, ethnicity, urban or rural residence, service connection, BMI (calculated from height and most recent weight value), military sexual trauma [20], physical and mental comorbidities, and VA site from CDW. Service connection refers to the development or aggravation of an illness or injury during military service [21]. Housing instability in the past 3 years was defined using a combination of VA CDW data, International Classification of Diseases-10 (ICD-10) codes and visit codes associated with homeless service use [22]. We created a dichotomous diabetes risk indicator (high risk = HbA1c 6.0-6.4% and/or history of gestational diabetes). Our models included some of the most common comorbidities among women Veterans [13, 23, 24]. Mental health conditions included depression, anxiety, post-traumatic stress disorder (PTSD), and substance use disorder. Each condition was constructed as a dichotomous variable, then these variables were aggregated to form a count of nine conditions for physical health (range 0–9) and a count of four conditions for mental health (range 0–4). An additional file lists ICD-10 codes used in our analysis [see Additional file 1].

### Analyses

We conducted bivariate analyses using t-tests for continuous variables and chi-square tests for categorical variables. We used logistic regression to examine the association between DPP enrollment and use of any VA preventive service for weight management or diabetes prevention. We adjusted for age, race, ethnicity, urban/rural residence, BMI, high diabetes risk, history of military sexual trauma, housing instability, count of physical and mental comorbidities, number of DPP recruitment contacts, and site. Analyses were performed using Stata version 18 (College Station, TX).

## Results

Our analytic cohort included a total of 1473 women Veterans who received DPP outreach at six VA sites between April 2021 and March 2024. On average, the mean age of women Veterans was 53 years (range 20–96), BMI was 34 kg/m^2^ (± 6 kg/m^2^) and average HbA1c was 5.9% (± 0.2%). Our sample included 0.7% Asian, 44% Black, 2% Hispanic, and 44% White women Veterans. Additionally, 41% lived in rural or highly rural neighborhoods and the majority (82%) were service connected, with an average service connection of 70% (± 28.6%). One-third (34%) of women Veterans reported prior military sexual trauma. Over half (58%) had a documented mental health diagnosis (e.g., depression, anxiety, substance use disorder or PTSD) and hypertension and hyperlipidemia were common (41.6% and 43.2%, respectively). An additional file details specific diagnoses included in our comorbidity counts by enrollment status [see Additional file 2]. Housing instability occurred in 8% of our sample within the last 3 years.

Half (50%) previously utilized VA weight management or diabetes prevention services. Within the last 3 years, 37.6% of women attended Whole Health visits, 14.4% attended MOVE! Clinic, 3.0% used metformin, and 2.2% used a weight loss medication.

Only 15.8% of women Veterans contacted for recruitment enrolled in the virtual DPP program. Compared to the 1241 women who did not enroll in DPP, enrollees (*n* = 232) were younger (51 years vs. 54 years), were contacted more frequently for recruitment (4 contacts vs. 2 contacts), and had higher levels of VA service connection (75% vs. 69%). Table 1 includes patient characteristics by enrollment.

**Table 1:** Characteristics of DPP cohort by enrollment status (*n* = 1473)

	Not Enrolled	Enrolled	*p*-value
N (%)	1241 (84.3%)	232 (15.8%)	
Age (mean years, SD)	53.84 (12.37)	50.70 (10.83)	**< 0.001**
Number of Recruitment Contacts	1.97 (1.18)	4.09 (1.59)	**< 0.001**
Race			
White	580 (46.7%)	71 (30.6%)	**< 0.001**
Black	526 (42.4%)	123 (53.0%)	
Unknown or Other (NHOPI, Asian, AIAN, multi-racial)	135 (10.9%%)	38 (16.4%)	
Ethnicity			
Non-Hispanic/Latino	1150 (92.7%)	203 (87.5%)	**0.011**
Urban vs. Rural			
Urban	716 (57.7%)	151 (65.1%)	**0.036**
Rural/Highly Rural	525 (42.3%)	81 (34.9%)	
Service Connection	1001 (80.7%)	205 (88.4%)	**0.005**
Service Connection Percentage (%, IQR)	69.17 (40.0)	75.23 (40.0)	**0.007**
Housing Instability	105 (8.5%)	14 (6.0%)	0.24
BMI (kg/m^2^, SD)	33.89 (5.99)	34.15 (6.24)	0.55
HbA1c (%, SD)	5.91 (0.20)	5.92 (0.19)	0.39
High Risk (HbA1c 6.0–6.4% or history of gestational diabetes)	733 (59.1%)	148 (63.8%)	0.19
Military Sexual Trauma	427 (34.4%)	80 (34.5%)	1.00
Number of Mental Comorbidities	1.09 (1.10)	1.24 (1.16)	0.063
Number of Physical Comorbidities	1.09 (0.98)	1.03 (0.96)	0.36
Outcome of Interest: Utilization of Prior VA Preventive Services			
Composite Outcome of Any Below	548 (44.2%)	136 (58.6%)	**< 0.001**
MOVE! Visit	171 (13.8%)	41 (17.7%)	0.13
Whole Health Visit	525 (42.3%)	131 (56.5%)	**< 0.001**
Nutrition Visit	938 (75.6%)	173 (74.6%)	0.74
Metformin or Weight Loss Drug Use	57 (4.6%)	15 (6.5%)	0.24

^a^NHOPI refers to Native Hawaiian or Pacific Islander, AIAN refers to American Indian or Alaska Native, BMI refers to Body Mass Index, and HbA1c refers to Hemoglobin A1c. Hispanic/Latino and unknown ethnicity were not reported in this table to ensure protected health information and patient identities remain confidential. Bolding indicates a *p*-value of < 0.05

In our adjusted models, prior use of VA preventative services was not significantly associated with DPP enrollment. However, younger women (OR:0.97, 95%CI 0.96–0.99) and those who received more recruitment contacts (OR:2.63, 95%CI 2.31–2.98) were significantly more likely to enroll in DPP. Women who experienced housing instability were significantly less likely to enroll (OR:0.44, 95%CI 0.21–0.92). Table 2 includes our adjusted results.

**Table 2:** Adjusted results

Enrolled	Odds Ratio	95% CI	*P*>|z|
Prior Use of VA Services	1.16	0.77, 1.75	0.469
Age	0.97	0.96, 0.99	**0.002** ^**b**^
# of Recruitment Contacts	2.63	2.32, 2.99	**< 0.001**
Race (ref: White)			
1. Black	0.93	0.54, 1.60	0.801
2. Other Race	0.77	0.28, 2.13	0.612
3. Unknown	1.39	0.63, 3.07	0.412
Ethnicity (ref: not Hispanic/Latino)			
1. Hispanic/Latino	0.99	0.39, 2.50	0.978
2. Unknown	2.43	1.08, 5.49	**0.032**
Urban vs. Rural (ref: Urban)			
1. Rural/Highly Rural	0.71	0.47, 1.07	0.098
Housing Instability	0.44	0.21, 0.92	**0.029**
BMI	0.98	0.95, 1.02	0.311
High Risk for Diabetes	0.97	0.65, 1.43	0.862
Military Sexual Trauma	0.84	0.56, 1.26	0.400
# of Physical Comorbidities	1.10	0.89, 1.34	0.378
# of Mental Comorbidities	1.02	0.86, 1.21	0.785

^b^Bolding indicates a P>|z| of < 0.05

## Discussion

Our study found no association between virtual DPP enrollment and women Veterans’ prior use of VA weight management and preventative services, including MOVE! Clinic, Whole Health visits, nutrition appointments, and use of metformin or weight-loss medications. These findings suggest that virtual DPP enrollment rates were similar among women Veterans with and without prior use of VA preventive services. Thus, virtual DPP may fill an existing gap for women Veterans who have never previously engaged in VA preventive services as well as those who have previously engaged in VA-based preventive services. We also found that women Veterans with housing instability were significantly less likely to enroll in virtual DPP. To our knowledge, we are the first study to assess the association between housing instability and DPP enrollment. Our findings underscore the importance of social determinants of health (SDOH) which are critical for healthcare access and use [25]. Further research examining the impact of housing instability and other SDOH on participation in preventive services within and outside of the VA is critically needed.

Our finding that younger women are more likely to enroll in virtual DPP aligns with prior studies showing that younger women may have a stronger preference for virtual DPP as compared with older women, who are more likely to prefer in-person DPP [26]. It is possible that virtual DPP provides a better “fit” for younger women who may be more likely to have competing demands for time, including work, caregiver roles or transportation barriers. In some, but not all, prior studies, there has been a positive correlation between BMI and DPP enrollment [8, 16, 26]. We found no association between BMI and DPP enrollment.

Though DPP is a well-established evidence-based intervention, enrollment in DPP remains low nationally, with multiple prior studies citing rates of enrollment under 10% [9, 16, 27, 28]. In a recent nationally representative analysis of diabetes prevention services, only 3% of people with prediabetes enrolled in a diabetes prevention program [29]. In our study, 15% of women who received contact enrolled in virtual DPP, and as noted above, this is higher uptake than what has been previously reported for other VA-based weight management services. Possible explanations for higher enrollment rates in virtual DPP include, but are not limited to, the virtual format of the program [15], a sense of community and social connection among women Veterans, and growing trust in the VA system. Veterans have previously perceived virtual DPP as a more convenient and time-effective option [15, 30, 31]. A prior study also found that some women Veterans preferred gender-tailored DPP delivery [30]. Based on these prior studies, EMPOWER 2.0 sites implemented virtual DPP with closed online women-only groups led by trained women coaches for the core phase of the program which may have contributed to higher enrollment rates.

Both within and out of the VA, additional efforts to help enhance DPP engagement among adults at risk of developing T2D are critically needed. Since diabetes is projected to be one of the top five leading causes of death by 2030 for adults in high-income countries, prevention is increasingly important [2]. Studies are needed to help identify more effective DPP recruitment strategies. We found that the number of recruitment contacts is associated with higher odds of enrollment. Other important strategies, such as direct physician referral, targeted community outreach through flyers or in-person recruitment, and electronic outreach through online advertisements or emails have also been previously cited [32]. Prior research on effective recruitment strategies among women Veterans is limited but emphasizes the importance of a peer advisor to establish collaboration, a strong relationship with the referring healthcare provider, and recruitment materials focused on highlighting the background of team members involved in the intervention [33, 34].

### Limitations

Our study has a few limitations. First, our study only included women Veterans from six VA sites, which limits the generalizability. However, our cohort was racially and ethnically diverse and representative of both rural and urban residents. Our sample included VA sites from the southeastern region of the United States which unfortunately have the highest rates of diabetes, obesity and associated healthcare disparities [35]. While we were able to uniquely include housing instability, we did not have access to other social determinants of health variables including income, employment status, or educational attainment. While more recruitment contacts were important for DPP enrollment, we do not have detailed information on the content and procedures for contact calls and potential variations that may have existed among sites. Each site was responsible for recruiting participants from their region, so site staff were able to tailor recruitment calls and materials as needed. Also, our analysis focused on women who made were aware that virtual DPP was an option at their VA site (i.e., we excluded women who may have met DPP eligibility criteria but did not receive outreach and therefore did not have an opportunity to enroll). As with any retrospective data analysis, our findings do not provide evidence for any causal relationships.

## Conclusions

Our study found that women Veterans who were younger, those who received more recruitment contacts, and those with stable housing were more likely to enroll in a virtual DPP that was implemented at six VA sites between 2022 and 2024. Prior use of VA weight management and preventive services was not associated with increased enrollment, suggesting that DPP may fill an existing gap, serving as both a complement to existing services and as an introductory preventive lifestyle program for women who have not previously engaged in VA-based services. Our study also suggests that utilization of recruitment strategies that increase the number of contacts with potential participants may be associated with increased enrollment in preventative services. Virtual DPP is an accessible and flexible option for women Veterans and our findings support continued delivery of this evidence-based intervention in the VA.

### Electronic supplementary material

Below is the link to the electronic supplementary material.

Supplementary Material 1

Supplementary Material 2

## Data Availability

Final data sets underlying this publication cannot be shared outside of the VA due to VA policies.

## Abbreviations

BMI: body mass index
CDC: Centers for Disease Control and Prevention
CDW: VA Corporate Data Warehouse
DPP: diabetes prevention program
EHR: electronic health record
EMPOWER: Enhancing Mental and Physical Health of Women through Engagement and Retention
GDM: gestational diabetes mellitus
HbA1c: hemoglobin A1c
ICD-10: International Classification of Diseases – 10
PTSD: post-traumatic stress disorder
QUERI: Quality Enhancement and Research Initiative
SDOH: social determinants of health
T2D: type 2 diabetes
VA: Department of Veterans Affairs
